# Supplementary material for: High-Fructose Diet Alters Intestinal Microbial Profile and Correlates with Early Tumorigenesis in a Mouse Model of Barrett’s Esophagus
Source: Microorganisms. 2021 Nov 25;9(12):2432. doi: 10.3390/microorganisms9122432 (PMC8708753; doi:10.3390/microorganisms9122432)
Supplement: Supplementary file 1 [file microorganisms-09-02432-s001.zip › microorganisms-1466088-supplementary.pdf]

microorganisms

MDPI

Supplementary Methods:

Tissue Preparation and Disease Evaluation

After euthanasia, the mice were dissected, and removed organs were subjected to downstream processing. The stomach was opened along the large curvature and flattened for documentation. Pictures were taken for subsequent performance of macroscopic scoring. Each stomach and esophagus was evaluated for tumor coverage, individual tumor size, and total tumor size and summed for an overall macroscopic score, as previously described [8,86]. The macroscopic tumor lesion score was determined as a mean of the tumor size and coverage score of the BE regions in cardia and esophagus. Scores were determined based on the following criteria: tumor size score 0 = no abnormalities, 1 = tumors <0.5 mm, 2 = tumors <1 mm, 3 = tumors <2 mm, 4 = tumors <3 mm; tumor coverage score: 0 = no abnormalities, 1 = <20% tumors, 2 = 20–50% tumors, 3 = >50–80% tumors, 4 = >80% tumors. Mouse tissues were fixed in formalin and paraffin embedded, then cut and stained with Hematoxylin and Eosin (H&E). Histopathology scoring was performed by an experienced gastroenterologist. Histopathologic scores were assigned using an established scoring system with previously established criteria for the influx of immune cells per high-power field, metaplasia, and dysplasia in the BE area at the SCJ [3], Inflammation was scored by the percentage of immune cell infiltration. Metaplasia was evaluated by the abundance of cells per gland and the abundance of glands with mucus-producing cells. Dysplasia was evaluated by the amount of cellular atypia and the presence of low- or high-grade dysplasia in single or multiple glands. The ratio of goblet-like cells, further referred to as goblet cells (GC), was estimated as a percentage of GCs in the BE region. Mucus production was assessed by PAS staining and quantified as a percentage of PAS-positive area in BE regions.

Immunohistochemistry

Standard immunohistochemical procedures with citrate buffer antigen retrieval (H-3300, Vector Labs, Burlingame, CA, USA) were performed with the following antibodies: Caspase-1 (E3914, Spring Bioscience, Pleasanton, CA, USA); 1:50, 30 min, room temperature), yH2AX (Ser139, #9718, Cell Signaling, 1:750, overnight, 4 °C), and Ki67 (AB15580, Abcam, Cambridge, CB2 0AX, UK); 1:2000, overnight, 4 °C). Quantification was accessed as the percentage of positive cells in the BE region, which were defined as the region between squamous epithelium and oxyntic mucosa of the stomach.

In Situ Hybridization

For the Lgr5 in situ hybridization, the RNAscope® 2.5 HD—Detection reagent BROWN (ACD, Newark, CA, USA), Mm-Lgr5 target probe (Cat. No. 312171, ACD, Newark, CA, USA), and all related reagents from ACD were used. The procedure was performed according to the manufacturer's protocol.

Stool Sampling Methods

Fecal samples were obtained from 31 mice over time (3–4 and 6 months) and at endpoints (6 or 9 months). From the 31 mice, 21 were HFrD fed and 10 were CD fed. Mice were fixed by hand and gently massaged in the lower abdomen to stimulate defecation. Fecal pellets were then collected with sterile tweezers, transferred directly to sterile tubes, snap-frozen in liquid nitrogen, and transferred to −80 °C for long-term storage until DNA isolation. For the collection of stool samples at endpoint, mice were euthanized, and fecal samples were directly taken from the distal part of the colon using an aseptic technique, snap-frozen in liquid nitrogen, and transferred to −80 °C for long-term storage until DNA isolation. To control for artifacts and ensure reproducibility between runs, two negative controls (a PCR control without template DNA and a DNA extraction control of 600 µL

stool stabilizer without stool), as well as a positive control using a mock community (No. D6300, ZymoBIOMICS, Irvine, CA, USA) were included in every batch of 45 samples.

High-Throughput Sequencing of 16S rRNA Gene Amplicons

DNA isolation was performed following a modification of the protocol by Godon et al. [16]. The fecal samples were transferred into a 2 mL screw-cap tube containing 0.1 mm silica beads and 600 µL DNA stool stabilizer (Invitek, Berlin, DE-BE, Germany). After adding 250 µL 4 M guanidinium thiocyanate and 500 µL 5% N-lauroylsarcosine sodium salt, samples were incubated at 70 °C and 700 rpm for 60 min (Eppendorf Themomixer comfort, Hamburg, DE-HH, Germany). Microbial cells were disrupted using the FastPrep-24 fitted with a CoolPrep adapter. For removing fecal contaminants, 15 mg polyvinylpyrrolidone were added. After vortexing, samples were centrifuged for 3 min at 15,000× *g* and 4 °C. Supernatant was transferred to a new 2 mL tube, the centrifugation step was repeated, and 500 µL of the supernatant was transferred into a new 2 mL tube. Five microliters RNase (10 mg/mL) were added, and samples were incubated at 37 °C and 700 rpm for 20–40 min. Pursuing isolation, DNA was purified by a silica-membrane approach, according to manufacturer's instructions (NucleoSpin gDNA Clean-up-Kit, 740230.250 Machery-Nagel, Düren, NRW, Germany). Nucleic acid concentration of purified DNA was measured with the NanoDrop 2000 spectrophotometer (Thermo Fisher Scientific, Waltham, MA, USA). The V3/V4 region of 16S rRNA genes was amplified from 12 ng of metagenomic DNA using the bacteria-specific primers 341F and 785R (25 cycles), following a two-step procedure to limit amplification bias [16]. Amplicons were purified using the AMPure XP system (Beckman Coulter, Brea, CA, USA). Purified amplicons were pooled in an equimolar amount and sequenced in paired-end modus (PE275) using a MiSeq system (Illumina, Inc., San Diego, CA, USA), following the manufacturer's protocol. 16S rRNA gene downstream analyses, including normalization, analysis of alpha- and beta-diversity, taxonomic abundance, and correlation analyses were performed using the Rhea pipeline created for the R-interface RStudio [106–108].

Sample Preparation for Metabolomic Analysis

The same sample extraction protocol was used for untargeted and targeted metabolomic analyses. Feces and cardia were weighed in 2 mL bead beater tubes (CKMix 2 mL, Bertin Technologies, Montigny-le-Bretonneux, Yvelines, France) filled with ceramic beads (1.4 mm and 2.8 mm ceramic beads i.d.). Samples were later normalized to input weight. Samples were homogenized using a bead beater (Precellys Evolution, Bertin Technologies, Montigny-le-Bretonneux, Yvelines, France) supplied with a Cryolys cooling module (Bertin Technologies, Montigny-le-Bretonneux, Yvelines, France) cooled with liquid nitrogen for 3 × 20 s at 10,000 rpm with 15 s breaks with 1 mL methanol as extraction solvent. The suspension was centrifuged for 10 min at 8000 rpm at 10 °C using an Eppendorf Centrifuge 5415R (Eppendorf, Hamburg, DE-HH, Germany). For metabolomic analysis of the serum samples, 30 µL of serum was extracted with 270 µL of the methanol. The leftover supernatant was stored at −80 °C for potential further analysis. An amount of 0.5 mL clear supernatant was transferred to a LC-MS/MS vial for untargeted measurement.

Metabolomic Analysis

The untargeted analysis was performed using a Nexera UHPLC system coupled with a TripleTOF6600 Q-TOF mass spectrometer (Sciex, Darmstadt, DE-HE, Germany), with sample separation by a HILIC UPLC BEH Amide 2.1 × 100, 1.7 µm analytic column with 400 µL/min flow rate and a reversed-phase Kinetex XB-C18, 2.1 × 100, 1.7 µm analytic column. Samples were measured in a randomized order. The mobile phase for the HILIC separation was 5 mM ammonium acetate in water (eluent A) and 5 mM ammonium acetate in acetonitrile/water (95/5, *v*/*v*) (eluent B). The gradient profile was 100% B from 0

to 1.5 min, 60% B at 8 min and 20% B at 10 min to 11.5 min, and 100% B at 12 to 15 min. The mobile phase for the reversed-phase separation was 0.1% formic acid (eluent A) and 0.1% formic acid in acetonitrile (eluent B) with 300 µL/min flow rate. The gradient profile was 0.2% B from 0 to 0.5 min to 100% B at 10 min, which was held for 3.25 min. Afterward, the column was equilibrated to starting conditions. A volume of 5 µL per sample was injected for both HILIC and RP chromatographic methods. The autosampler was cooled to 10 °C and the column oven-heated to 40 °C. A quality control (QC) sample, which was pooled from all samples, was injected after every 10th sample. Samples were measured in information-dependent acquisition (IDA) mode. MS settings in the positive mode were as follows: Gas 1 55, Gas 2 65, Curtain gas 35, Temperature 500 °C, Ion Spray Voltage 5500 V, and declustering potential 80 V. The mass range of the TOF MS and MS/MS scans were 50–2000 m/z, and the collision energy was ramped from 15 to 55 V. MS settings in the negative mode were as follows: Gas 1 55, Gas 2 65, Cur 35, Temperature 500 °C, Ion Spray Voltage −4500, and declustering potential −80. The mass range of the TOF MS and MS/MS scans were 50–2000 m/z, and the collision energy was ramped from −15 to −55 V. Results were visualized via the web-based analysis tool "XCMS-Viewer" of the BayBioMS. Preprocessing of data via the XCMS viewer was performed by first converting raw data to an open format via the ProteoWizard platform [109]. Peak-picking, alignment, correspondence performed using the peak-density method, and peak-grouping were performed using the previously published R-package "xcms" available via the Bioconductor website for bioinformatic processing [110–112]. The ion mass of the features was compared with different databases (MS1 annotation), including the MS-DIAL DB, HMDB, PubChem, and in-house reference DBs at BayBioMS, allowing some common modifications and a tolerance window of mass (15 ppm). The cosine distance between measured and reference spectra was calculated (MS2 annotation). Significant enrichment of the metabolites in the groups was defined as a mean difference (mean diff.) −2≤ and ≥2, represented as log10 mean diff. −0.3≤ and ≥0.3 with a p-value of ≤0.05, represented as log10 *p*-value of ≥1.3. Detected metabolites were evaluated for correct annotation by reviewing chromatographic characteristics between DB and measured metabolites or by availability of data of previously measured reference standards.

For the quantitation of short-chain fatty acids (SCFAs), the 3-NPH method was used [28]. Briefly, 40 µL of the fecal, cardia, or serum extract and 15 µL of isotopically labeled standards (ca 50 µM) were mixed with 20 µL 120 mM EDC HCl-6% pyridine-solution and 20 µL of 200 mM 3-NPH HCL solution. After 30 min at 40 °C and shaking at 1000 rpm using an Eppendorf Thermomix (Eppendorf, Hamburg, DE-HH, Germany), 900 µL acetonitrile/water (50/50, *v*/*v*) was added. After centrifugation at 13000 U/min for 2 min, the clear supernatant was used for analysis. SCFA analysis was performed using a QTRAP 5500 mass spectrometer (Sciex, Darmstadt, DE-HE, Germany) coupled with an ExionLC AD (Sciex, Darmstadt, DE-HE, Germany) ultrahigh performance liquid chromatography system. The electrospray voltage was set to −4500 V, curtain gas to 35 psi, ion source gas 1 to 55 psi, ion source gas 2 to 65 psi, and the temperature to 500 °C. The MRM parameters were optimized using commercially available standards for the SCFAs. The chromatographic separation was performed on a 100 × 2.1 mm, 100 Å, 1.7 µm, Kinetex C18 column (Phenomenex, Aschaffenburg, DE-BY, Germany) column with 0.1% formic acid (eluent A) and 0.1% formic acid in acetonitrile (eluent B) as elution solvents. An injection volume of 1 µL and a flow rate of 0.4 mL/min were used. The gradient elution started at 23% B, which was held for 3 min; afterward, the concentration was increased to 30% B at 4 min, with another increase to 40% B at 6.5 min; at 7 min, 100% B was used, which was held for 1 min, and at 8.5 min, the column was equilibrated at starting conditions. The column oven was set to 40 °C and the autosampler to 15 °C. Data acquisition and instrumental control were performed with Analyst 1.7 software (Sciex, Darmstadt, Germany).

Transcriptional Profile Analysis

Total RNA was isolated from SCJ, liver, and colon using the Maxwell 16 LEV simplyRNA Tissue Kit (AS1280, Promega, Walldorf, DE-BW, Germany) and the Maxwell® 16 machine, according to manufacturer's instructions. Tissue samples (approximately 30 mg, 1–2 mm^2 was thawed on ice and homogenized in 1 mL of RTL buffer (Lysis buffer, 1015762; Qiagen, Hilden, DE-NRW, Germany) with 1% β-Mercaptoethanol (M3148, Sigma, Taufkirchen, DE-BY, Germany) for approximately 30 s using the SilentCrusher M (Heidolph, Schwabach, DE-BY, Germany) and an ultrasonic device (Bandelin Sonoplus, Berlin, DE-BE, Germany), if necessary. RNA was extracted from the homogenate and eluted in 40 µL nuclease-free water. RNA concentration and quality were determined on a Nanodrop 2000 spectrophotometer (Thermo Scientific, Waltham, MA, USA).

Reverse Transcription and Quantitative Real-Time Polymerase Chain Reaction (qRT-PCR) Analysis

The reverse transcription of RNA (250 ng) into cDNA was performed using the QuantiTect Reverse Transcription Kit (205314, Qiagen, Hilden, DE-NRW, Germany), following the manufacturer's protocol. Previous to the reverse transcription, the RNA was incubated in gDNA wipeout buffer for 2 min at 42 °C for elimination of gDNA. qRT-PCR analysis with QuantiFast SYBR Green PCR Kit (4000) (204057, Qiagen, Hilden, DE-NRW, Germany) was performed on the LightCycler® 480 II (Roche, Basel, Switzerland). The reactions were performed in duplicates, and the Thermocycler conditions used were: (1) 95 °C for 3 min and (2) 40 cycles of 95 °C for 30 s, 55 °C for 30 s, and 72 °C for 30 s. Primer sequences of targeted genes were selected from previously published literature [113,114] and ordered as high-purity salt-free (HPSF) lyophilizates from Sigma Aldrich (Taufkirchen, DE-BY, Germany). Primers of the housekeeping genes (Glyceraldehyde 3-phosphate dehydrogenase and Cyclophilin A) were used to normalize the RNA levels of the target genes. All primers were re-evaluated for gene specificity by blasting on Primer-Blast (NCBI). Primers were dissolved in PCR-grade water to 100 mM stock solutions and diluted 1:10 to working solutions of 10 mM. The primers for Aldolase B were forward, 5′-AATAGGGACCAGCCCATTCT -3′, and reverse, 5′- CTTCCAGCCTTGCTATCCAA -3′ The primers for Triokinase were forward, 5′- GTGTTTGCCTCTCCTCCTGT -3′, and reverse, 5′- AGCCGATCCCCAGTGTAGTT -3′. The primers for Glucose-6-Phosphatase were forward, 5′- GGCTCACTTTCCCCATCAGG -3′, and reverse, 5′-ATCCAAGTGCGAAACCAAACAG -3′. The primers for Hexokinase were forward, 5′-ATGTGTGTGCCACTCCAGAC -3′, and reverse, 5′- GACCCGGAAGTTTGTTCCTC -3′. The primers for Phosphofructokinase were forward, 5′-CATGAATGCAGCTGTGCGCTCC -3′, and reverse, 5′-CCAGCCCACTTCTTGCACCTGA -3′. The primers for Fructose Bisphosphatase were forward, 5′- AGCCTTCTGAGAAGGATGCTC -3′, and reverse, 5′-GTCCAGCATGAAGCAGTTGAC -3′. The primers for Glycerinaldehyd-3-Phosphat-Dehydrogenase were forward, 5′- GACATCAAGAAGGTGGTGAAGCA -3′, and reverse, 5′- ATACCAGGAAATGAGCTTGACAAA -3′. The primers for Cyclophilin A were forward, 5′- ATGGTCAACCCCACCGTGT -3′, and reverse, 5′-TTCTGCTGTCTTTGGAACTTTGT -3′.

Flow Cytometry

After dissection, tissues were shredded to small pieces with scissors and digested with freshly prepared digestion buffer for 30 min at 37 °C and 150–250 rpm in a shaking incubator (TH15, Edmund Bühler GmbH, Bodelshausen, DE-BW, Germany). Digestion buffer for esophageal tissue consisted of Krebs-Ringer Solution + BSA 40 mg/mL (Roth) + Collagenase P 2 mg/mL (Roche, Basel, BS, Switzerland). Digestion buffer for cardia, forestomach, and the rest of the stomach consisted of RPMI (Thermo Fisher Scientific, Waltham, MA, USA) + Collagenase P 2 mg/mL (Roche, Basel, BS, Switzerland) + Pronase

2 mg/mL (Roche, Basel, BS, Switzerland). Digestion buffer for liver and colon consisted of RPMI (Thermo Fisher Scientific, Waltham, MA, USA) + Collagenase P 2 mg/mL (Roche, Basel, BS, Switzerland). After digestion, tissues were passed through 40 μm cell strainers (352340, FALCON; 542040, Greiner Bio-One, Kremsmünster, AT-4, Austria) with the sterile end of syringe plungers (4606108V, B Braun, Meldingen, DE-HE, Germany). Cells were centrifuged for 10 min at 4 °C and 400× *g*. Supernatants were discarded, and samples were resuspended in 200–300 μL FACS washing buffer, transferred to a U-bottom 96 well cell culture plate with lid (650 180, Greiner Bio-One, Kremsmünster, AT-4, Austria), and centrifugated at 4 °C and 400× *g* for 10 min. After disposal of the supernatant, samples were washed again with FACS washing buffer. Supernatant was disposed and samples were incubated with 50 μL of the staining solutions for 30 min in the dark. Following the staining, cells were washed twice with FACS washing buffer. Remaining cell pellets were resuspended in 200 μL FACS washing buffer and transferred to 1.4 mL flow cytometry tubes (MP32022, MICRONIC, Chattanoga, TN, USA). Fluorescence-activating cell sorting data were acquired on a Gallios flow cytometer (Beckman Coulter, Brea, CA, USA), transferred to the FlowJo™ Software (Becton, Dickinson, and Company, Franklin Lakes, NJ, USA) and processed using compensation and gating templates previously generated. Cell populations are represented as the percentage of cells compared with all CD45+ cells. Following antibodies were used for the Myeloid cell staining: CD11c FITC (11-0114-85), F4/80 APC (17-4801-82), CD11b APC eFluor 780 (47-0112-82), CD45 eFluor 450 (48-0451-82), Ly-6C PE (12-5932-82), and Ly-6G Alexa Flour 700 (56-5931-82). Following antibodies were used for the T cell staining: NK1.1 APC eFluor 780 (47-5941-82), CD3e FTIC (11-0033-82), CD8a APC (17-0081-82), CD4 eFluor 450 (48-0041-82), γδ TCR PE (12-5711-82), CD69-Alexa700 (104539), CD25 PE Cy7 (25-0251-82), and CD45 PerCP Cy5 (45-0451-82); 7-AAD was used to quantify live cells. All antibodies were purchased from eBioscience, San Diego, CA, USA).

RNA-Sequencing

Library preparation for bulk sequencing of poly(A)-RNA was carried out, as described previously [115]. Briefly, barcoded cDNA of each sample was generated with a Maxima RT polymerase (Thermo Fisher Scientific, Waltham, MA, USA) using oligo-dT primer-containing barcodes, unique molecular identifiers (UMIs), and an adaptor. Ends of the cDNAs were extended by a template switch oligo (TSO), and full-length cDNA was amplified with primers binding to the TSO site and the adaptor. NEB UltraII FS kit (New England BioLabs, Ipswich, MA, USA) was used to fragment cDNA. After end repair and A-tailing, a TruSeq adapter was ligated and 3′-end-fragments were finally amplified using primers with Illumina P5 and P7 overhangs. In comparison with the reference method [115], P5 and P7 sites were exchanged to allow sequencing of the cDNA in read1 and barcodes and UMIs in read2 to achieve a better cluster recognition. The library was sequenced on a NextSeq 500 (Illumina Inc., San Diego, CA, USA) with 63 cycles for the cDNA in read1 and 16 cycles for the barcodes and UMIs in read2.

Gencode gene annotations M25 and the mouse reference genome GRCm38 were derived from the Gencode homepage (EMBL-EBI). Drop-Seq tools v1.12 [116] were used for mapping raw sequencing data to the reference genome. The resulting UMI filtered count matrix was imported into R v4.0.5. Counts per million (CPM) values were calculated for the raw data, and genes with a mean CPM value less than 1 were removed from the dataset. Prior to differential expression analysis with DESeq2 v1.18.1 [117], dispersion of the data was estimated with a parametric fit using a univariate model, where diet was specified as the independent variable. The Wald test was used for determining differentially regulated genes between diets, and shrunken log2 fold changes were calculated afterward. A gene was determined as differentially regulated if the absolute apeglm shrunken log2 fold change was at least 1 and the adjusted p-value was below 0.01. GSEA v4.0.3 [118] was performed in the weighted pre-ranked mode, where the apeglm shrunken foldchange was used as the ranking metric. All genes tested for differential

expression were used for GSEA analysis with gene sets from MsigDB v7.4 [119,120]. A pathway was considered to be significantly associated with a diet if the FDR value was below 0.05. Rlog transformation of the data was performed for visualization and further downstream analysis. Leading edge genes of selected pathways from the GSEA are displayed as heatmap. Heatmap shows z-transformed expression data.

Supplementary Figures:

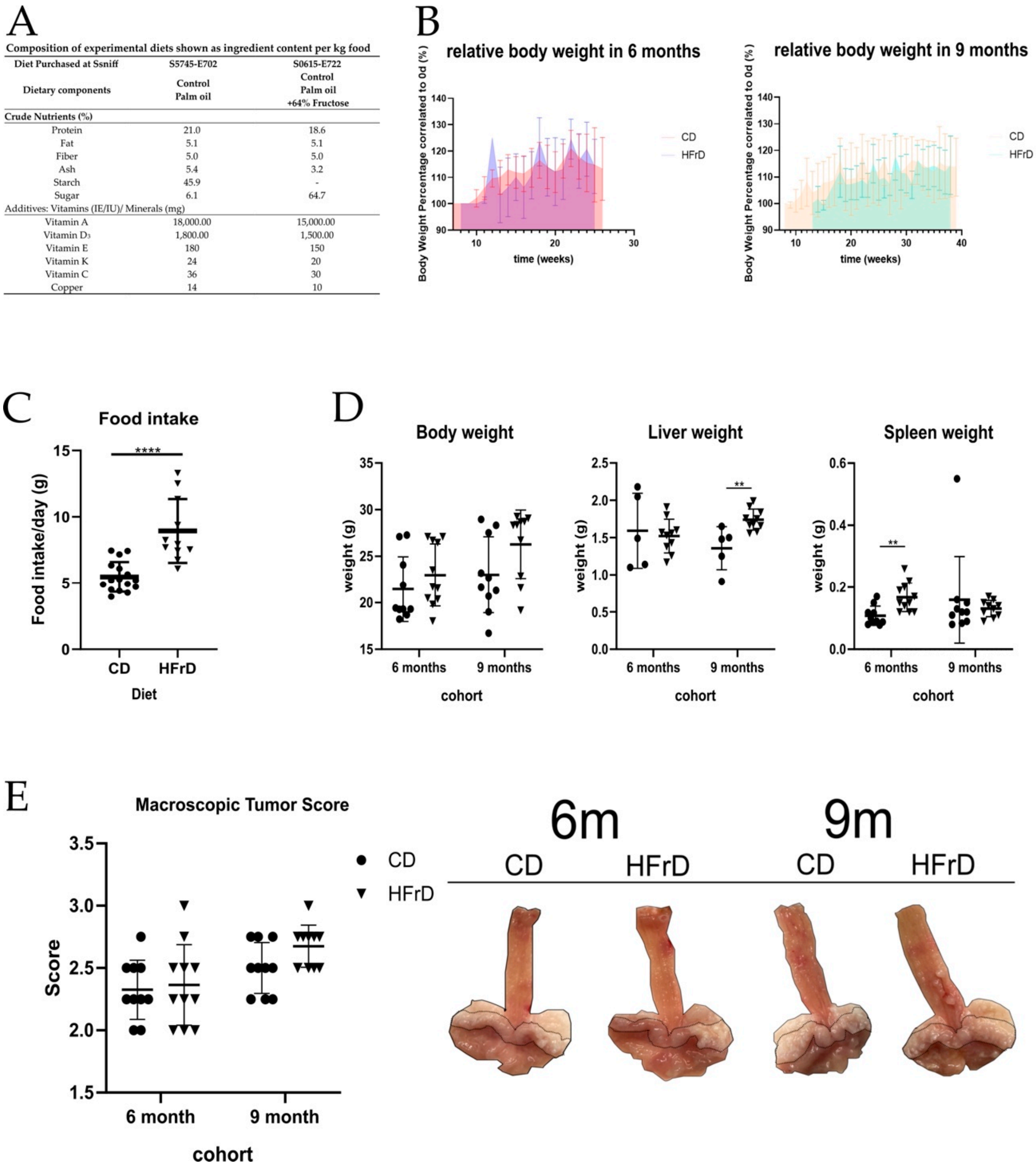
Composition of experimental diets shown as ingredient content per kg food

Diet Purchased at Ssniff	S5745-E702	S0615-E722
Dietary components	Control Palm oil	Control Palm oil +64% Fructose
Crude Nutrients (%)		
Protein	21.0	18.6
Fat	5.1	5.1
Fiber	5.0	5.0
Ash	5.4	3.2
Starch	45.9	-
Sugar	6.1	64.7
Additives: Vitamins (IE/IU)/ Minerals (mg)		
Vitamin A	18,000.00	15,000.00
Vitamin D_3	1,800.00	1,500.00
Vitamin E	180	150
Vitamin K	24	20
Vitamin C	36	30
Copper	14	10

Figure S1. Diet, dietary preferences, body-, liver- and spleen-weight, and macroscopic tumor score. (**A**) Composition of experimental diets shown as ingredient content per kg food; (**B**) Relative body weight (%) over time from 6 month- and 9 month-old mice fed with HFrD or CD. Body weight was measured weekly and is represented as percentage from the weight at the beginning of the dietary intervention; (**C**) Comparison of food consumption between intervention groups. Food intake was monitored every 3 to 4 days and is represented as daily food consumption. On the left, all values to food intake including loss of food through changes in food consistency. The daily food intake was significantly higher ($p_{A,B} = < 0.0001$); (**D**) Weight of whole body, liver and spleen. While the body weight did not differ significantly between treatment groups, the weight of the liver increased significantly in HFrD mice compared to CD mice at an age of 9 months (*p*

= 0.0037). Additionally, the weight of the spleen was significantly higher in HFrD mice at an age of 6 months (p = 0.0027); (**E**) Macroscopic Tumor Score (MTS) – combined MTS from cardia and esophagus, and macroscopic pictures. In both cohorts, the MTS did not significantly differ between the intervention groups. (**C**–**E**): Statistical comparison between intervention groups and age cohorts. Data are represented as mean ± SD. Depending on the normal distribution of the data, either an unpaired t test or a Mann-Whitney test was used for the statistical analysis. (**C**–**E**): circles represent samples from CD-fed mice and triangles represent samples from HFrD-fed mice. Significance level: ** (very significant); **** (extremely significant).

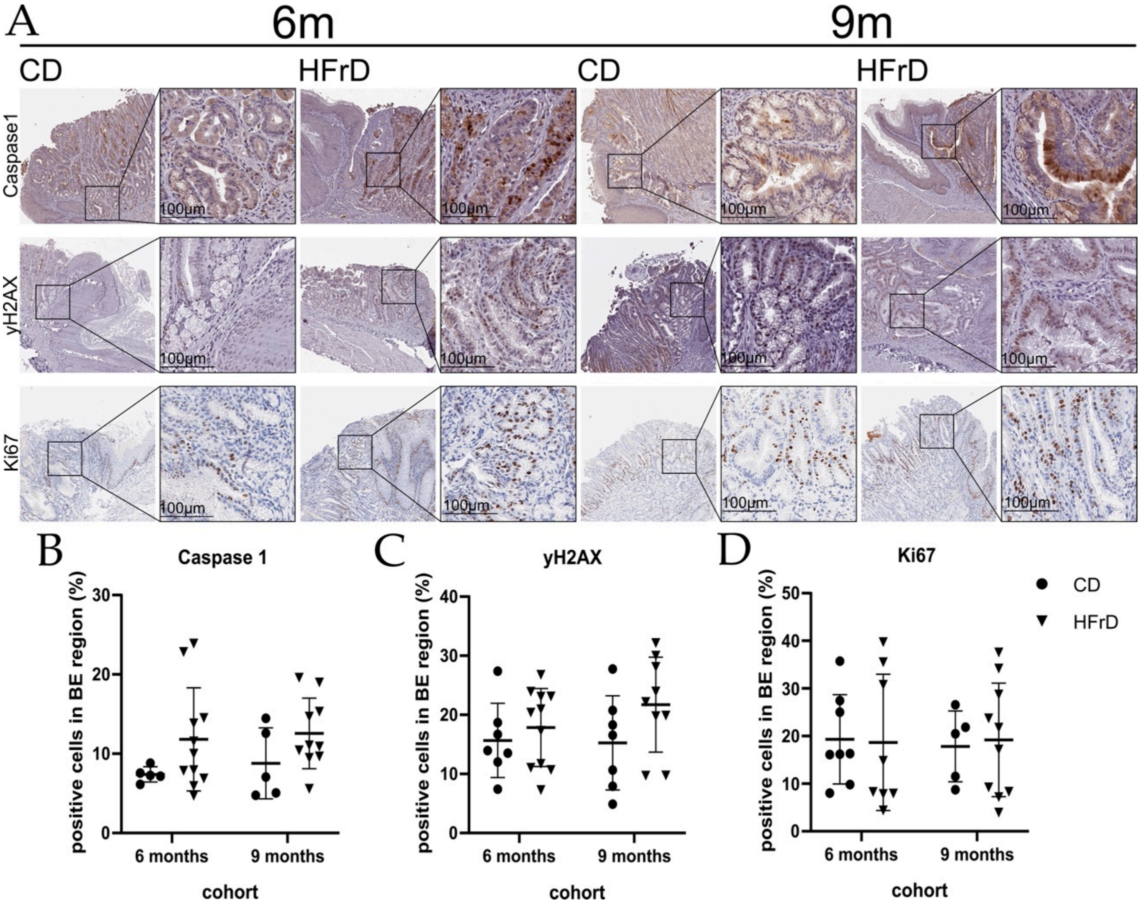

Figure S2. Caspase1-, yH2AX- and Ki67-positive cells in the BE region. (**A**) Microscopic pictures; (**B**) Analysis of the area of Caspase1-positive cells in the BE-region. While the area of Caspase1-positive cells was lesser in CD mice than in HFrD mice, the difference was not significant in neither the 6-month- nor the 9-month-cohort; (**C**) Analysis of yH2AX-positive cells in the BE-region. While the percentage of yH2AX-positive cells was higher in HFrD-fed mice than in CD-mice of both cohorts, the difference was not significant. (**D**) Analysis of Ki67-positive cells in the BE region. The content of Ki67-positive cells did not differ between the intervention groups and age cohorts. (**B**–**D**): statistical comparison between age cohorts and intervention groups. Data are represented as mean ± SD. Depending on the normal distribution of the data, either an unpaired t test or a Mann-Whitney test was used for the statistical analysis. (**B**–**D**): circles represent samples from CD-fed mice and triangles represent samples from HFrD-fed mice.

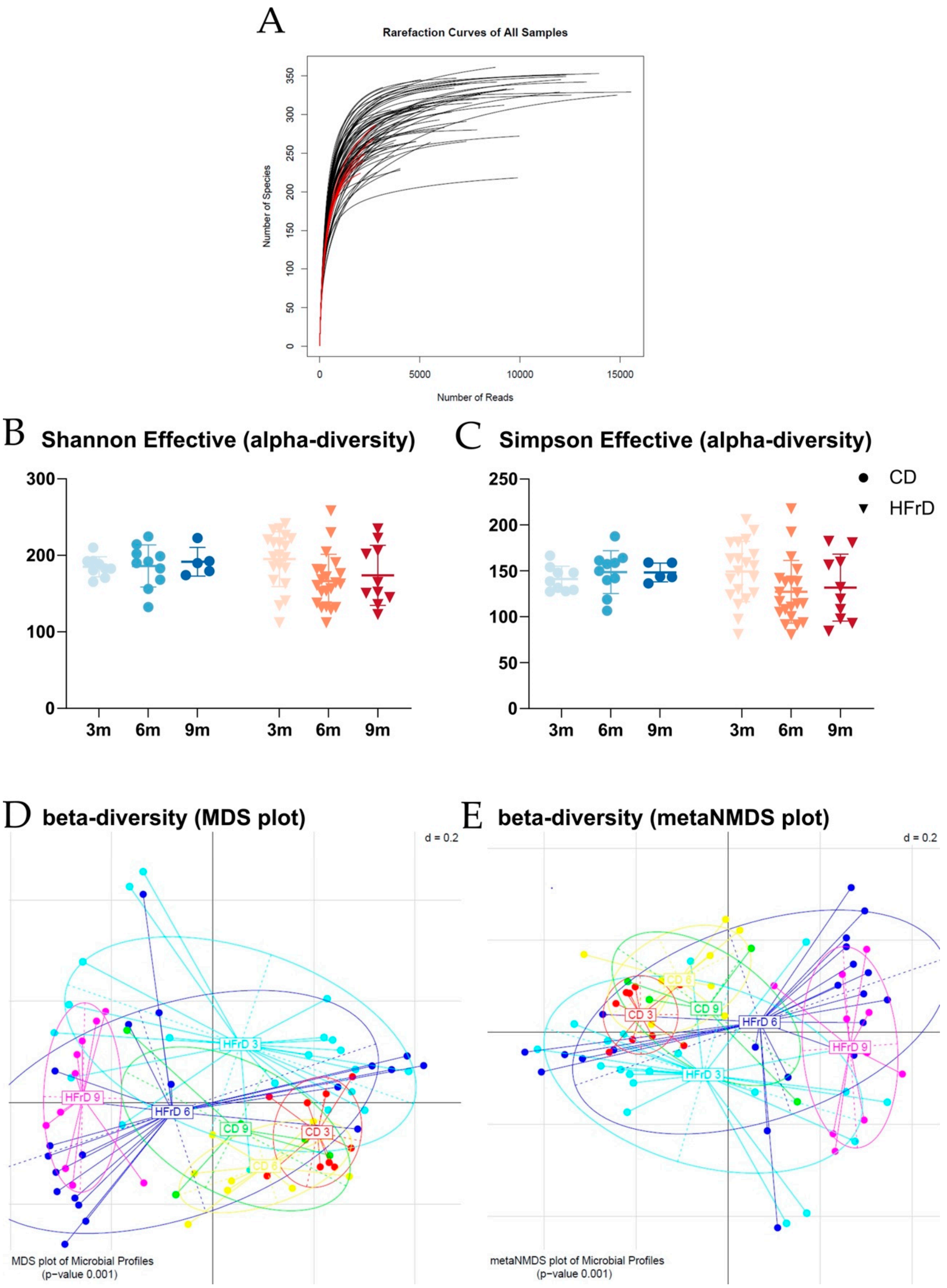

Figure S3. Further details of microbiome characterization. (**A**) Rarefaction curves of all samples; (**B**) Shannon Effective Index (alpha-diversity); (**C**) Simpson Effective Index (alpha-diversity); (**D**) graphical representation of beta-diversity as MDS plot; (**E**) graphical representation of beta-diversity as metaNMDS plot.

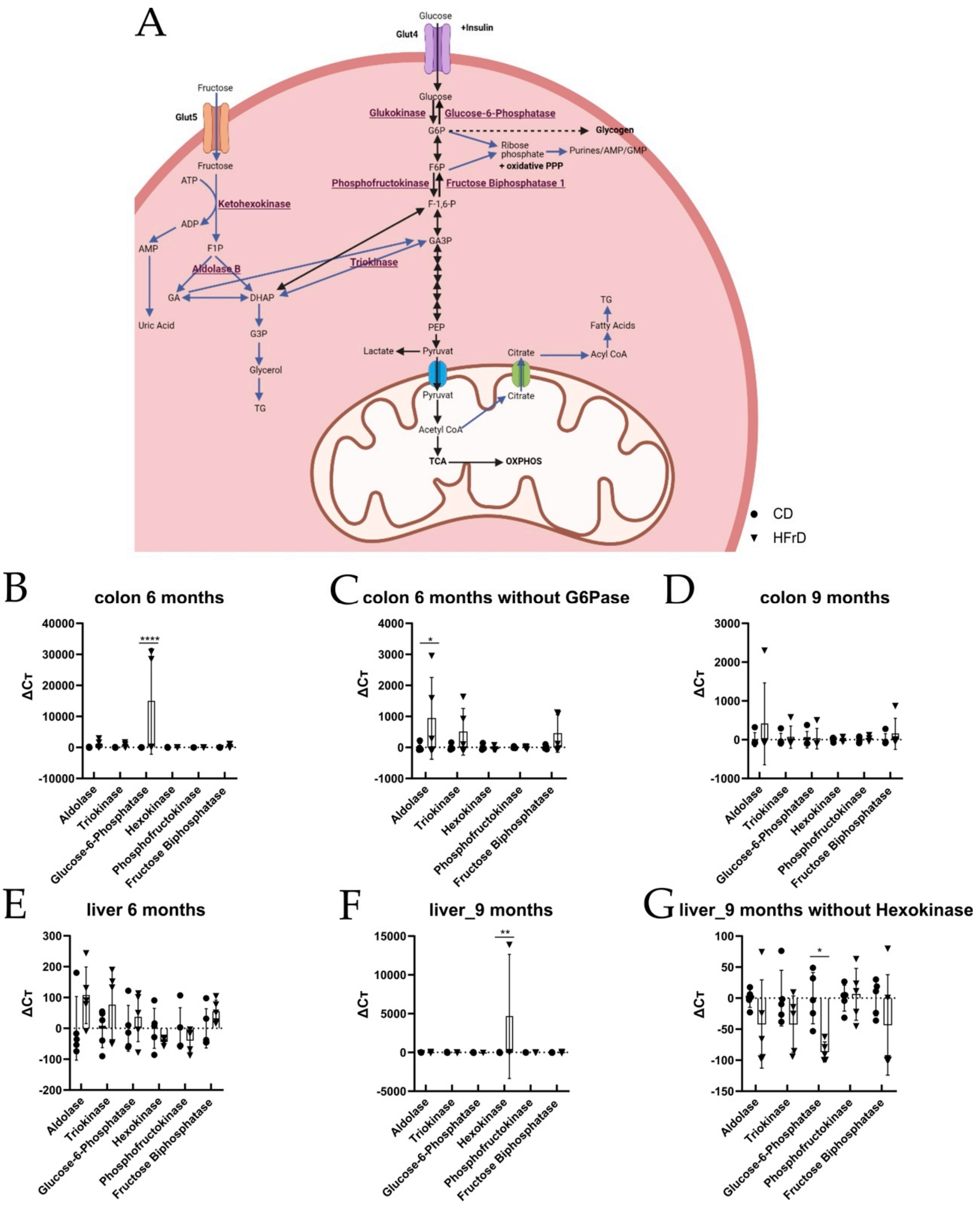

Figure S4. Simplified graphical representation of the fructose and glucose metabolism and relative expression of enzymes relevant for fructose metabolism. (**A**) Unlike glucose that enters the cell via GLUT4, the diffusion of fructose occurs in an insulin-independent way through GLUT5. Black arrows represent reactions of the glycolysis. Purple arrows represent reactions mainly driven as consequence of the fructose metabolism that can however, also be driven as response to the glucose degradation. Fructose-1-phosphate (F1B), glyceraldehyde (GA), dihydroxyacetone phosphate (DHAP), glucose-6-phosphate (G6P), fructose-6-phosphate (F6P), fructose-1,6 biphosphate (F-1,6-P), glycerol-3-phosphate (G3P), glyceraldehyde-3-phosphate (GA3P), phosphoenolpyruvate (PEP), triglyceride (TG), tricarboxylic acid cycle (TCA), adenosine triphosphate (ATP), adenosine diphosphate (ADP), adenosine monophosphate (AMP), guanosine monophosphate (GMP), pentose phosphate pathway (PPP), oxidative phosphorylation (OXPHOS) – Figure adapted from [11,104,121–123] and created in BioRender.com. Enzyme expression in: (**B**) the colon of 6-month-old mice, (**C**) the colon of 6-month-old mice without outlier (Glucose-6-Phosphatase), (**D**) the colon of 9-month-old mice, (**E**) the liver of 6-month-old mice, (**F**) the liver of 9-month-old mice, and (**G**) the liver of 9-month-old mice without outlier (Hexokinase); statistical comparison between intervention groups. While in the colon of 6-month-old HFrD-fed mice the expression of Glucose-6-Phosphatase ($p = < 0.0001$) and Aldolase ($p = 0.0342$) increased significantly in comparison to CD-fed mice of the same age

cohort, no significant differences were observed in the colon of 9-month-old mice. While in the liver of 9-month-old HFrD-fed mice the Hexokinase increased significantly (*p*= 0.0025) and the Glucose-6-Phosphatase decreased significantly (p= 0.0238) in comparison to the CD-fed mice, no significant differences were observed in the liver of the 6-month-cohort. Data are represented as mean + SD. For the statistical analysis a 2way ANOVA with Sidak´s multiple comparisons test was performed. (**C**–**E**): circles represent samples from CD-fed mice and triangles represent samples from HFrD-fed mice. Significance level: * (significant); ** (very significant); **** (extremely significant).

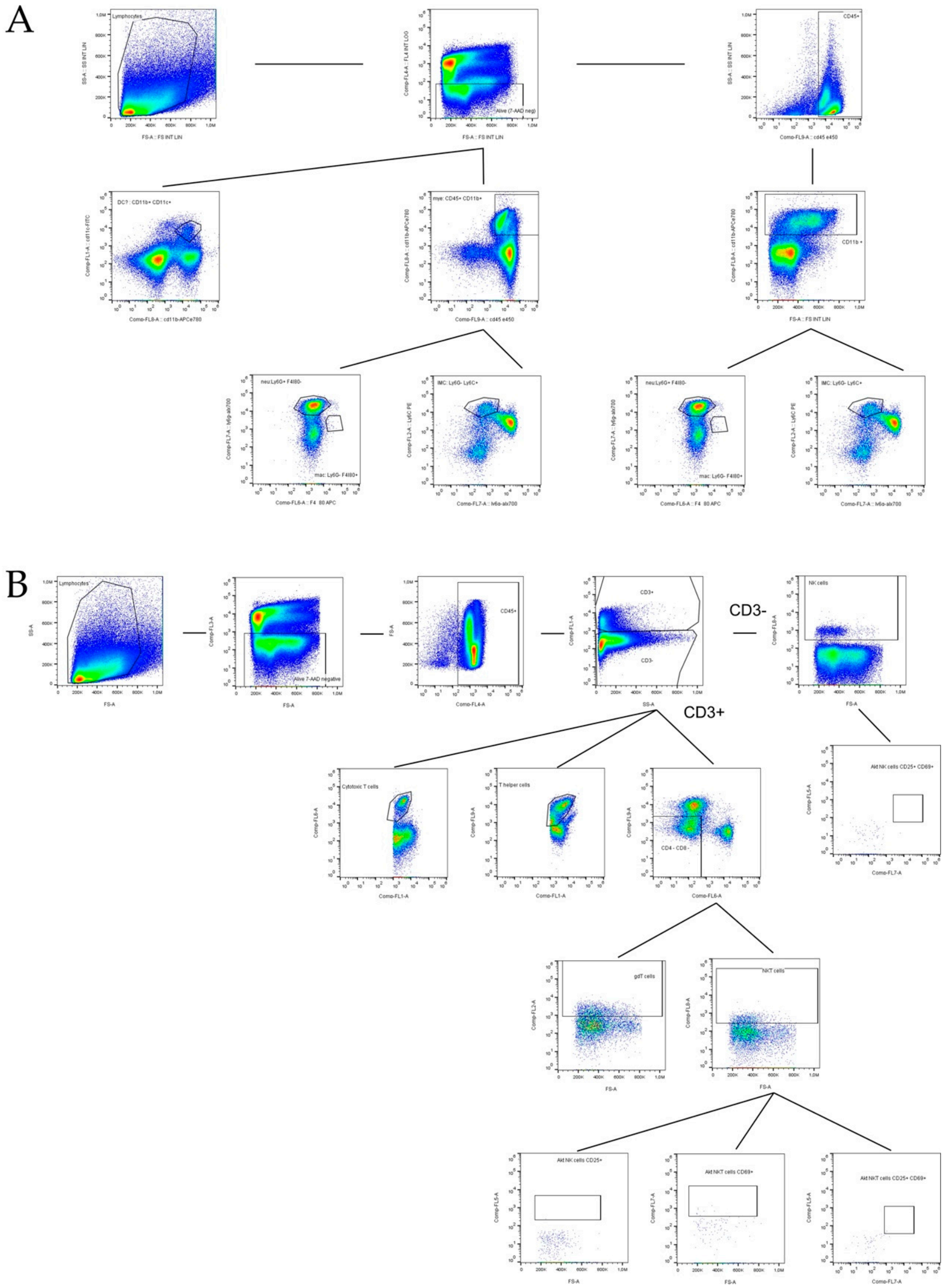

Figure S5. Gating flowchart of (**A**) myeloid cell populations and (**B**) T cell populations. (**A**) Living cells were gated for (1) CD45 positivity, (2) CD45+ CD11b+ and (3) CD11b+ CD11c+, and were then used for the further identification of immune cells. Ly6G+ and F4|80- cells were identified as neutrophils; Ly6G- and F4|80+ cells were identified as macrophages; Ly6G-

and Ly6C+ cells were identified as immature myeloid cells and; CD11b+ and CD11c+ cells were identified as dendritic cells. (**B**) Living cells were gated for CD45 positivity and then for CD3 positivity and negativity. CD3- cells were gated for NK1.1 positivity. Positive cells were referred to as NK cells and were further gated for active NK cells (CD25+ and CD69+). CD3 positive cells were then gated for CD4 and CD8 load. CD4+ cells were considered T helper cells and CD8+ cells as cytotoxic T cells. For the identification of γδ T cells and NKT cells, CD4- and CD8- cells were gated for γδ TCR and NK1.1 positivity. Identified NKT cells were further gated for active NKT cells (CD25+, CD60+ and, CD25+ and CD60+).

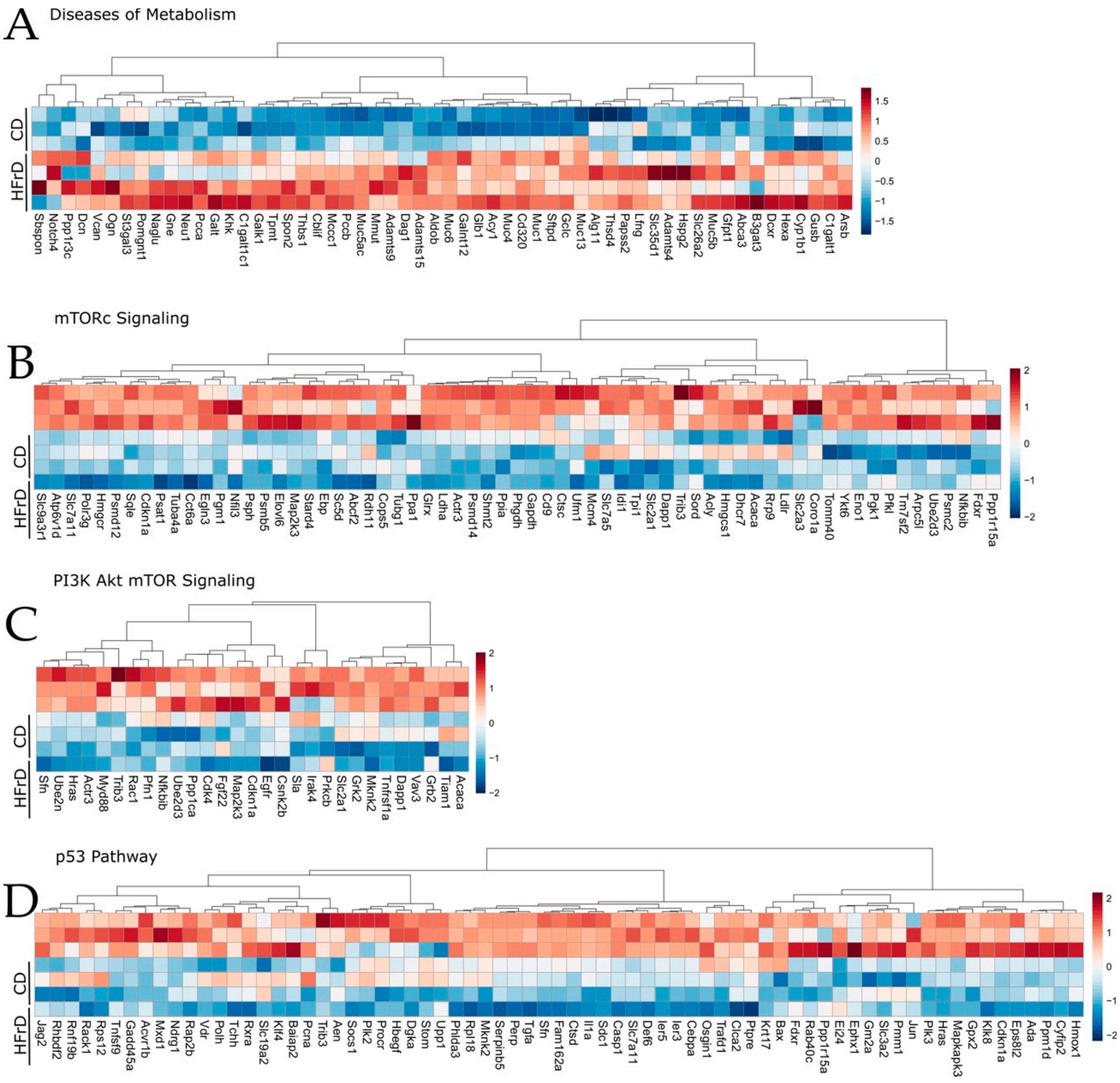

Figure S6. Heatmaps of metabolic signaling enriched in HFrD mice and pro-oncogenic genesets in CD identified by GSEA. (**A**) Significantly enriched Geneset in HFrD mice (**B**–**D**) Significantly enriched Genesets in CD mice depicted as heatmaps showing gene expression changes among samples.

References

106. Lagkouvardos, I.; Fischer, S.; Kumar, N.; Clavel, T. Rhea: a transparent and modular R pipeline for microbial profiling based on 16S rRNA gene amplicons. *PeerJ* **2017**, *5*, e2836, doi:10.7717/peerj.2836.
107. Team, R. *RStudio: Integrated Development Environment for R*, RStudio, Inc.: Boston, 2016.
108. R Core Development Team *R: A language and environment for statistical computing*, R Foundation for Statistical Computing: Vienna, 2018.
109. Chambers, M.C.; Maclean, B.; Burke, R.; Amodei, D.; Ruderman, D.L.; Neumann, S.; Gatto, L.; Fischer, B.; Pratt, B.; Egertson, J. A cross-platform toolkit for mass spectrometry and proteomics. *Nature biotechnology* **2012**, *30*, 918-920.
110. Smith, C.A.; Want, E.J.; O'Maille, G.; Abagyan, R.; Siuzdak, G. XCMS: processing mass spectrometry data for metabolite profiling using nonlinear peak alignment, matching, and identification. *Analytical chemistry* **2006**, *78*, 779-787.

111. Tautenhahn, R.; Böttcher, C.; Neumann, S. Highly sensitive feature detection for high resolution LC/MS. *BMC bioinformatics* **2008**, *9*, 1-16.
112. Benton, H.P.; Want, E.J.; Ebbels, T.M. Correction of mass calibration gaps in liquid chromatography–mass spectrometry metabolomics data. *Bioinformatics (Oxford, England)* **2010**, *26*, 2488-2489.
113. Kebede, M.; Favaloro, J.; Gunton, J.E.; Laybutt, D.R.; Shaw, M.; Wong, N.; Fam, B.C.; Aston-Mourney, K.; Rantzau, C.; Zulli, A.; et al. Fructose-1,6-bisphosphatase overexpression in pancreatic beta-cells results in reduced insulin secretion: a new mechanism for fat-induced impairment of beta-cell function. *Diabetes* **2008**, *57*, 1887-1895, doi:10.2337/db07-1326.
114. Patel, C.; Douard, V.; Yu, S.; Tharabenjasin, P.; Gao, N.; Ferraris, R.P. Fructose-induced increases in expression of intestinal fructolytic and gluconeogenic genes are regulated by GLUT5 and KHK. *Am J Physiol Regul Integr Comp Physiol* **2015**, *309*, R499-509, doi:10.1152/ajpregu.00128.2015.
115. Parekh, S.; Ziegenhain, C.; Vieth, B.; Enard, W.; Hellmann, I. The impact of amplification on differential expression analyses by RNA-seq. *Scientific Reports* **2016**, *6*, 25533, doi:10.1038/srep25533.
116. Macosko, E.Z.; Basu, A.; Satija, R.; Nemesh, J.; Shekhar, K.; Goldman, M.; Tirosh, I.; Bialas, A.R.; Kamitaki, N.; Martersteck, E.M.; et al. Highly Parallel Genome-wide Expression Profiling of Individual Cells Using Nanoliter Droplets. *Cell* **2015**, *161*, 1202-1214, doi:10.1016/j.cell.2015.05.002.
117. Love, M.I.; Huber, W.; Anders, S. Moderated estimation of fold change and dispersion for RNA-seq data with DESeq2. *Genome Biol* **2014**, *15*, 550, doi:10.1186/s13059-014-0550-8.
118. Subramanian, A.; Tamayo, P.; Mootha, V.K.; Mukherjee, S.; Ebert, B.L.; Gillette, M.A.; Paulovich, A.; Pomeroy, S.L.; Golub, T.R.; Lander, E.S.; et al. Gene set enrichment analysis: A knowledge-based approach for interpreting genome-wide expression profiles. *Proceedings of the National Academy of Sciences* **2005**, *102*, 15545, doi:10.1073/pnas.0506580102.
119. Liberzon, A.; Subramanian, A.; Pinchback, R.; Thorvaldsdóttir, H.; Tamayo, P.; Mesirov, J.P. Molecular signatures database (MSigDB) 3.0. *Bioinformatics* **2011**, *27*, 1739-1740, doi:10.1093/bioinformatics/btr260.
120. Liberzon, A.; Birger, C.; Thorvaldsdóttir, H.; Ghandi, M.; Mesirov, J.P.; Tamayo, P. The Molecular Signatures Database (MSigDB) hallmark gene set collection. *Cell Syst* **2015**, *1*, 417-425, doi:10.1016/j.cels.2015.12.004.
121. Dewdney, B.; Roberts, A.; Qiao, L.; George, J.; Hebbard, L. A Sweet Connection? Fructose's Role in Hepatocellular Carcinoma. Biomolecules **2020**, *10*, doi:10.3390/biom10040496.
122. Christen, P.; Jaussi, R.; Benoit, R. Glykolyse und Citratzyklus. In Biochemie und Molekularbiologie: Eine Einführung in 40 Lerneinheiten, Christen, P., Jaussi, R., Benoit, R., Eds.; Springer Berlin Heidelberg: Berlin, Heidelberg, **2016**; pp. 161−175.
123. Lee, H.-J.; Cha, J.-Y. Recent insights into the role of ChREBP in intestinal fructose absorption and metabolism. BMB reports **2018**, *51*, 429−436, doi:10.5483/BMBRep.2018.51.9.197.
